# Meta-analysis on blood transcriptomic studies identifies consistently coexpressed protein–protein interaction modules as robust markers of human aging

**DOI:** 10.1111/acel.12160

**Published:** 2013-11-19

**Authors:** Erik B van den Akker, Willemijn M Passtoors, Rick Jansen, Erik W van Zwet, Jelle J Goeman, Marc Hulsman, Valur Emilsson, Markus Perola, Gonneke Willemsen, Brenda WJH Penninx, Bas T Heijmans, Andrea B Maier, Dorret I Boomsma, Joost N Kok, Pieternella E Slagboom, Marcel JT Reinders, Marian Beekman

**Affiliations:** 1 Department of Molecular Epidemiology, Leiden University Medical CenterPO Box 9600, 2300 RC, Leiden, The Netherlands; 2 The Delft Bioinformatics Lab, Delft University of TechnologyPO Box 5031, 2600 GA, Delft, The Netherlands; 3 Department of Psychiatry, VU University Medical Center, Neuroscience Campus Amsterdam, VU University Medical CenterA.J. Ernststraat 1187, 1081 HL, Amsterdam, The Netherlands; 4 EMGO Institute for Health and Care Research, Neuroscience Campus AmsterdamVan der Boechorststraat 7, 1081 BT, Amsterdam, The Netherlands; 5 Department of Medical Statistics, Leiden University Medical CenterPO Box 9600, 2300 RC, Leiden, The Netherlands; 6 Icelandic Heart AssociationHoltasmari 1, IS-201, Kópavogur, Iceland; 7 National Institute for Health and WelfarePO Box 30, 00271, Helsinki, Finland; 8 Department of Biological Psychology, VU UniversityVan der Boechorststraat 7, 1081 BT, Amsterdam, The Netherlands; 9 Section of Gerontology and Geriatrics, Department of Internal Medicine, VU University Medical CenterDe Boelelaan 1117, 1007 MB, Amsterdam, The Netherlands; 10 Department of Algorithms, Leiden Institute of Advanced Computer Science, University of LeidenNiels Bohrweg 1, 2333 CA, Leiden, The Netherlands; 11 Netherlands Consortium for Healthy Ageing, Leiden University Medical CenterPO Box 9600, 2300 RC, Leiden, The Netherlands

**Keywords:** aging, blood transcriptomics, meta-analysis, network-based analysis, protein–protein interactions

## Abstract

The bodily decline that occurs with advancing age strongly impacts on the prospects for future health and life expectancy. Despite the profound role of age in disease etiology, knowledge about the molecular mechanisms driving the process of aging in humans is limited. Here, we used an integrative network-based approach for combining multiple large-scale expression studies in blood (2539 individuals) with protein–protein Interaction (PPI) data for the detection of consistently coexpressed PPI modules that may reflect key processes that change throughout the course of normative aging. Module detection followed by a meta-analysis on chronological age identified fifteen consistently coexpressed PPI modules associated with chronological age, including a highly significant module (*P* = 3.5 × 10^−38^) enriched for ‘*T-cell activation*’ marking age-associated shifts in lymphocyte blood cell counts (*R*^2^ = 0.603; *P* = 1.9 × 10^−10^). Adjusting the analysis in the compendium for the ‘*T-cell activation*’ module showed five consistently coexpressed PPI modules that robustly associated with chronological age and included modules enriched for ‘*Translational elongation*’, ‘*Cytolysis*’ and ‘*DNA metabolic process*’. In an independent study of 3535 individuals, four of five modules consistently associated with chronological age, underpinning the robustness of the approach. We found three of five modules to be significantly enriched with aging-related genes, as defined by the GenAge database, and association with prospective survival at high ages for one of the modules including *ASF1A*. The hereby-detected age-associated and consistently coexpressed PPI modules therefore may provide a molecular basis for future research into mechanisms underlying human aging.

## Introduction

A steadily growing life expectancy of the general western population throughout the past two centuries (Oeppen & Vaupel, 2002) has imposed the urgency for understanding the adverse effects of aging for public health and its relation to the observed large variation in healthy lifespan (Hitt *et al*., 1999). Age-dependent detrimental processes strongly attenuate prospects for future health, with chronological age being the major risk factor for mortality and virtually all common diseases in the western world (Wilson *et al*., 1998). Aging is a systemic ailment marked by a gradual metabolic decline eventually leading to a state of senescence on both the cellular and organismal level that seems to be caused by the accumulation of damage over time (Kirkwood, 1977). Despite their profound role for disease etiology, the existing knowledge concerning the molecular mechanisms driving biological aging processes in humans is limited.

Construction of consistent age-associated signatures has proven to be challenging as a multitude of gene expression studies have identified age-associated genes so far, though with limited mutual overlap (Passtoors *et al*., 2008; de Magalhaes *et al*., 2009). This inconsistency is most likely due to variable technical circumstances, small study sizes, and low signal-to-noise ratios, typically observed when analyzing the aging transcriptome. More similarity was observed at the pathway level, across tissues and even species (Partridge & Gems, 2002; Zahn *et al*., 2006), suggesting that the analysis of the aging transcriptome by functionally grouped gene sets is a promising alternative for the classical individual-gene analyses.

Rather than employing literature-based sets of genes sharing similar biological functions, so-called network approaches are increasingly used, which infer functional clusters of genes from the expression data itself by exploiting gene coexpression patterns hidden within the data (Zhang & Horvath, 2005). Alternatively, changes in these gene coexpression patterns that occur with age might be used for inferring a functional grouping from the data (Southworth *et al*., 2009). However, coexpression patterns may contain spurious gene–gene correlations (Stuart *et al*., 2003), which makes the use of multiple data sources simultaneously or the integration with other additional information sources on functional relationships between genes desirable.

Established modulators of aging processes in model organisms were reported to spatially cluster within networks constructed of protein–protein interaction (PPI) data (de Magalhaes & Toussaint, 2004; Bell *et al*., 2009). Hence, PPI networks can be exploited for prioritizing new aging-associated genes (Witten & Bonchev, 2007; Tacutu *et al*., 2012) or for refining modules of coexpressed genes that are correlated during the course of aging (Xue *et al*., 2007). We previously demonstrated that the inference of these so-called coexpressed PPI modules has a high reproducibility across multiple expression datasets in breast cancer (van den Akker *et al*., 2011), and here we extend this algorithm to combine multiple gene expression datasets on aging.

Though many algorithms for network inference exist (Marbach *et al*., 2012), relatively little attention has gone to the problem of network inference and subsequent associations with a phenotype using multiple heterogeneous expression data sources simultaneously. Merging the expression data into a single set and using this for network inference clearly surpasses the differences in correlation structures present within each dataset. Irrespective of the type of network inference chosen, we propose to handle such heterogeneity by integrating the gene–gene similarity measures obtained across expression datasets using a suitable meta-analysis setting. Thus, in the approach described in this paper, we employ a meta-analysis for inferring a consistent gene–gene network that serves as a basis for identifying consistently coexpressed PPI modules, which are subsequently analyzed with respect to chronological age across datasets using again a meta-analysis.

To robustly characterize the changes of the blood transcriptome associated with chronological age, we have build a compendium using three large-scale transcriptomic studies (Goring *et al*., 2007; Emilsson *et al*., 2008; Inouye *et al*., 2010) generated in blood comprising 2539 individuals on which we applied our integrative network approach. For comparison, two types of individual-gene meta-analyses were performed as well, which in combination with an enrichment analysis yielded only broad terms for age-associated cellular processes. Application of our integrative network-based approach, yielded five consistently coexpressed PPI modules showing robust age associations and functional enrichments for *‘Translational elongation’*, *‘Cytolysis’* and *‘DNA metabolic process’*, which seem to reflect downstream mTOR signaling events or cell-cycle checkpoints. Finally, we show that four of five modules replicate in an independent cohort, and that they are enriched for known longevity- and aging-related genes and that the expression of one module associates with prospective survival at old age.

## Results

### The largest transcriptome compendium for normative aging

To robustly characterize the changes of the blood transcriptome throughout the course of normative aging in the range of 15–94 years, we built a gene expression compendium using three large-scale transcriptomic studies performed in blood: the San Antonio Family Heart Study (SAFHS) (Goring *et al*., 2007), the Icelandic Family Blood (IFB) cohort (Emilsson *et al*., 2008) and the Dietary, Lifestyle, and Genetic determinants of Obesity and Metabolic syndrome (DILGOM) study (Inouye *et al*., 2010). Data of IFB were measured in two roughly equally sized batches, from this point on referred to as IFB_A and IFB_B, and was treated as two separate datasets in the downstream analysis. Data quality was critically reassessed and reannotated yielding a compendium of 9047 unique genes expressed in 2539 individuals divided over four datasets (SAFHS: 1,240, IFB_A: 411, IFB_B: 435, DILGOM: 454) [Table 1 & Experimental procedures].

**Table 1:** Descriptives of the datasets composing the compendium

Study	Tissue	Cohort	Ethnicity	No. of start total†	No. of end total‡	No. of males (%)‡	Mean age‡	Min age‡	Max age‡
SAFHS§	Lymphocytes	San Antonio Family Heart Study	Mexican Americans (USA)	1240	1240	506 (40.8)	39.3	15	94
IFB_A	Peripheral blood	Icelandic Family Blood (IFB) cohort	Caucasian (Icelandic)	904¶	411	198 (48.2)	48.8	19	84
IFB_B	Peripheral blood	Icelandic Family Blood (IFB) cohort	Caucasian (Icelandic)	904¶	434	180 (41.5)	46.2	20	76
DILGOM	Peripheral blood	DILGOM (Dietary, Lifestyle, and Genetic determinants of Obesity and Metabolic syndrome)	Caucasian (Finnish)	518††	454	195 (43.0)	51.6	30	70

† Number of individuals with matching phenotypic data per study when obtained.

‡ Statistics computed after preprocessing.

§ Expression and phenotypic data were obtained from ArrayExpress under accessions: E-TABM-305.

¶ Data of IFB were measured in two batches. This figure indicates the total number of individuals before preprocessing or removal of duplicates across batches.

†† A small batch was detected and all samples belonging to it were removed.

### Limited overlap of age-associated genes between studies within the compendium

**Figure 1:**
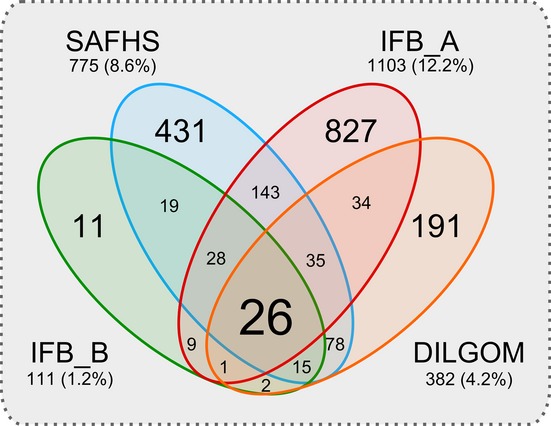
Significantly age-associated genes in studies of the blood compendium. A Venn analysis was performed for inspecting the overlap of the significantly age-associated genes found within different studies. The majority of the consistently detected age-associated genes (24 of 26) show a decreased expression with advancing age and include the following: *ARH*, *BACH2*, *CCR7*, *ECRG4*, *EDAR*, *EPHA1*, *EPHX2*, *FAM102A*, *FAM134B*, *FBLN2*, *FCGBP*, *FLNB*, *IL24*, *LRRN3*, *NELL2*, *NMT2*, *NRCAM*, *OXNAD1*, *PDE9A*, *PHGDH*, *PIK3IP1*, *SIRPB2*, *SUSD3,* and *TSGA14*. The remaining 2 consistently age-associated genes showing increased expressions are *ARP10* and *SYT11*. See Table S1 (Supporting information) for more details.

The most straightforward method for an integrative analysis across datasets is to first compute the age-association genes per dataset and subsequently inspect the overlap of significant results. A linear model adjusted for gender yielded between 111 (1.2%) and 1103 (12.2%) significantly age-associated genes per dataset (Bonferroni correction, α ≤ 0.05), of which 26 genes were significantly associated with age in all four datasets [Fig. 1 and Table S1, Supporting information]. These results confirmed the high discrepancy between lists of age-associated genes previously reported in literature, even though now observed in equal or similar tissues (Passtoors *et al*., 2008; de Magalhaes *et al*., 2009).

### Rank-based integration of age-associated genes improves consistency between studies

As repeatedly applied cutoffs across multiple heterogeneous datasets may lead to high false exclusion rates of age-associated genes, we investigated whether age-association rankings were consistently high across datasets by applying a rank integration approach (Breitling *et al*., 2004; de Magalhaes *et al*., 2009). From the 9047 genes present in the compendium, 247 consistently showed highly ranked differential expressions with age across the four datasets, of which 195 remained significant after permutation tests (both at FDR<=0.05) [Experimental procedures]. Of these 195 genes, 128 (65.6%) showed decreased and 67 (34.4%) showed increased expression levels with age. The top 25 genes with increased and decreased expression are displayed in Tables 2 and 3, respectively, and include many of the age-associated genes previously identified, like *LRNN3*, *LEF1*, *and SYT11* (Hong *et al*., 2008; Harries *et al*., 2011; Passtoors *et al*., 2012). Results for all 9047 genes in the compendium are provided in Table S2 (Supporting information).

**Table 2:** Top 25 genes according to the gene statistic (*U*_i_) having *increase*d expression with age

Symbol	GeneID	*P*-value*	q-value*	*P*-value**	q-value**
GPR56	9289	5.3 × 10^−09^	4.8 × 10^−05^	1.0 × 10^−06^	0.0018
HF1	3075	2.3 × 10^−08^	8.1 × 10^−05^	1.0 × 10^−06^	0.0018
SYT11	23208	2.7 × 10^−08^	8.1 × 10^−05^	≤ 5.0 × 10^−7^	0.0018
ARP10	164668	7.3 × 10^−08^	1.7 × 10^−04^	1.0 × 10^−06^	0.0018
B3GAT1 (CD57)	27087	1.1 × 10^−07^	2.0 × 10^−04^	3.0 × 10^−06^	0.0021
SLC1A7	6512	1.8 × 10^−07^	2.6 × 10^−04^	3.2 × 10^−05^	0.0110
IFNG	3458	5.0 × 10^−07^	6.4 × 10^−04^	1.1 × 10^−05^	0.0065
DSCR1L1	10231	6.1 × 10^−07^	6.8 × 10^−04^	2.0 × 10^−06^	0.0021
ARK5	9891	7.9 × 10^−07^	7.9 × 10^−04^	3.0 × 10^−06^	0.0021
PIG13	81563	9.3 × 10^−07^	8.8 × 10^−04^	1.0 × 10^−06^	0.0018
SPUVE	11098	1.1 × 10^−06^	8.8 × 10^−04^	1.2 × 10^−05^	0.0067
PDGFRB	5159	1.2 × 10^−06^	8.8 × 10^−04^	1.5 × 10^−06^	0.0021
EDG8	53637	1.4 × 10^−06^	9.4 × 10^−04^	7.8 × 10^−05^	0.015
MARLIN1	152789	1.5 × 10^−06^	9.4 × 10^−04^	5.0 × 10^−06^	0.0032
TGFBR3	7049	2.0 × 10^−06^	0.0012	2.8 × 10^−05^	0.011
GZMB	3002	2.4 × 10^−06^	0.0013	5.0 × 10^−04^	0.050
CX3CR1	1524	2.9 × 10^−06^	0.0014	2.9 × 10^−05^	0.011
STYK1	55359	3.3 × 10^−06^	0.0015	4.8 × 10^−05^	0.013
ADRB2	154	3.7 × 10^−06^	0.0016	3.0 × 10^−06^	0.0021
GAF1	26056	7.1 × 10^−06^	0.0029	7.2 × 10^−05^	0.015
CTSL	1514	7.7 × 10^−06^	0.0030	3.2 × 10^−04^	0.040
GFI1	2672	1.1 × 10^−05^	0.0040	3.0 × 10^−06^	0.0021
TTC38	55020	1.1 × 10^−05^	0.0040	7.6 × 10^−05^	0.015
AGPAT4	56895	1.2 × 10^−05^	0.0041	2.5 × 10^−06^	0.0021
GZMA	3001	1.4 × 10^−05^	0.0045	3.3 × 10^−04^	0.040

* *P*- and *q*-values determined using the gamma distribution of the gene statistic, *U*_i_.

** *P*- and *q*-values determined using permutation of the gene statistic, *U*_i_.

**Table 3:** Top 25 genes according to the gene statistic (*U*_i_) having *decrease*d expression with age

Symbol	GeneID	*P*-value*	q-value*	*P*-value**	q-value**
LRRN3	54674	1.3 × 10^−12^	1.2 × 10^−8^	≤ 5.0 × 10^−7^	3.2 × 10^−4^
FCGBP	8857	3.2 × 10^−10^	1.5 × 10^−6^	≤ 5.0 × 10^−7^	3.2 × 10^−4^
CCR7	1236	1.1 × 10^−9^	3.2 × 10^−6^	≤ 5.0 × 10^−7^	3.2 × 10^−4^
NELL2	4753	2.0 × 10^−8^	4.5 × 10^−5^	1.0 × 10^−6^	3.8 × 10^−4^
NRCAM	4897	3.1 × 10^−8^	5.6 × 10^−5^	≤ 5.0 × 10^−7^	3.2 × 10^−4^
IGJ	3512	1.5 × 10^−7^	2.3 × 10^−4^	2.6 × 10^−4^	0.019
LEF1	51176	1.9 × 10^−7^	2.5 × 10^−4^	≤ 5.0 × 10^−7^	3.2 × 10^−4^
FAM134B	54463	2.2 × 10^−7^	2.5 × 10^−4^	≤ 5.0 × 10^−7^	3.2 × 10^−4^
PACAP	51237	2.5 × 10^−7^	2.5 × 10^−4^	1.5 × 10^−6^	4.8 × 10^−4^
ITM2C	81618	2.8 × 10^−7^	2.5 × 10^−4^	3.5 × 10^−6^	8.1 × 10^−4^
PIK3IP1	113791	3.0 × 10^−7^	2.5 × 10^−4^	1.0 × 10^−6^	3.8 × 10^−4^
PDE9A	5152	5.1 × 10^−7^	3.8 × 10^−4^	1.0 × 10^−6^	3.8 × 10^−4^
BACH2	60468	6.9 × 10^−7^	4.8 × 10^−4^	1.0 × 10^−6^	3.8 × 10^−4^
FLJ12895	65982	9.5 × 10^−7^	6.0 × 10^−4^	1.5 × 10^−6^	4.8 × 10^−4^
FAM102A	399665	1.1 × 10^−6^	6.0 × 10^−4^	≤ 5.0 × 10^−7^	3.2 × 10^−4^
FBLN2	2199	1.1 × 10^−6^	6.0 × 10^−4^	≤ 5.0 × 10^−7^	3.2 × 10^−4^
FLNB	2317	1.2 × 10^−6^	6.0 × 10^−4^	≤ 5.0 × 10^−7^	3.2 × 10^−4^
APEG1	10290	1.2 × 10^−6^	6.0 × 10^−4^	1.0 × 10^−6^	3.8 × 10^−4^
EPHX2	2053	1.3 × 10^−6^	6.0 × 10^−4^	1.5 × 10^−6^	4.8 × 10^−4^
TNFRSF17	608	1.3 × 10^−6^	6.1 × 10^−4^	1.2 × 10^−4^	0.011
MYC	4609	1.6 × 10^−6^	6.6 × 10^−4^	3.5 × 10^−6^	8.1 × 10^−4^
NT5E	4907	1.7 × 10^−6^	6.6 × 10^−4^	1.0 × 10^−6^	3.8 × 10^−4^
TOSO	9214	1.7 × 10^−6^	6.6 × 10^−4^	1.0 × 10^−6^	3.8 × 10^−4^
ARH	26119	3.2 × 10^−6^	0.0012	2.0 × 10^−6^	6.2 × 10^−4^
OXNAD1	92106	3.3 × 10^−6^	0.0012	≤ 5.0 × 10^−7^	3.2 × 10^−4^

* *P*- and *q*-values determined using the gamma distribution of the gene statistic, *U*_i_.

** *P*- and *q*-values determined using permutation of the gene statistic, *U*_i_.

### Functional enrichments of individual-gene analysis are not informative for normative aging

We next identified enriched functional groupings among genes significantly associated with normative aging using DAVID focusing on GO_FAT terms. Whereas the 26 genes from the overlap did not yield any significantly enriched terms, the 195 significant genes obtained with the rank integration approach yielded 11 significant enriched groupings when run at default settings [Tables S3 and S4, Supporting information respectively]. Interestingly, enriched terms include *‘Glycosylation site:N-linked’* (*P* = 6.1 × 10^−5^, Benjamini corrected), previously linked to the inflamm-aging theory (Dall’olio *et al*., 2013). However, as most of the 11 identified terms are rather broadly defined, like *‘disulfide bond’* or *‘signal peptide’,* little detailed knowledge is gained on potential molecular mechanisms underlying normative aging following the individual-gene analysis approach.

### A novel integrative network approach for detecting consistent coexpressed PPI modules

To improve robustness against noise and increase power, we used a novel integrative network-based approach to explore functional age-associated groupings of genes. The proposed approach detects consistently coexpressed PPI modules across multiple datasets (for details see Experimental procedures and Data S1, Supporting information). Using the four transcriptomic datasets mapped onto the PPI network, we detected a total of 162 consistently coexpressed PPI modules ranging in size from 2 to 37 genes [see Fig. S1, Supporting information for a complete overview]. The following steps in our analysis were limited to the subset of 27 coexpressed PPI modules counting at least five genes. Application of DAVID yielded significant functional enrichments for 19 of the 27 identified coexpressed PPI modules [Table S5, Supporting information], suggesting that the applied approach grouped genes according to plausible biological functions.

### Age-associated coexpressed PPI modules point toward T-cell activation

**Figure 2:**
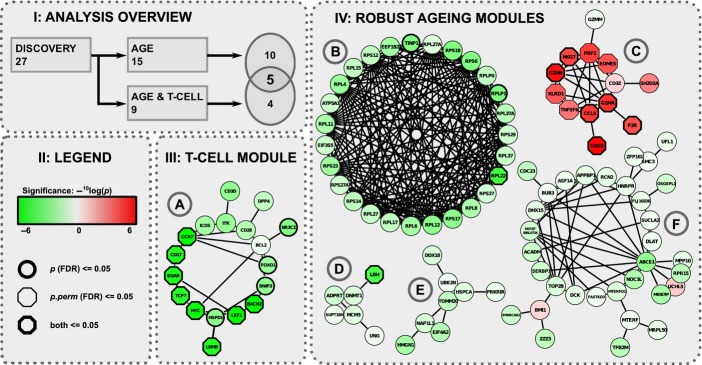
Overview main results of the integrative network-based approach. Panel 1: Overlap of the PPI network and cluster analysis of the transcriptomic data reveals 27 modules, 15 are significantly associated with age, 9 are significantly associated with age when corrected for the ‘T-cell activation’ module expression, and the 5 most robust findings are found in the overlap. Panel 2: Legend: Genes are represented by nodes, whose shape and color reflect the results of the individual-gene statistic (Ui). The red and green colors denote a correlating or anti-correlating relationship of gene expression with age, respectively. The intensity of the coloring indicates the significance of the gamma-distributed transformed rank product statistics. Nodes marked by a thick bordering or a hexagon shaped bordering represent genes with FDR adjusted P-values ≤ 0.05 for respectively the analytical and permutation-based approach. Panel 3: The coexpressed PPI module that is enriched for ‘T-cell activation’. Panel 4: B-F: 5 coexpressed PPI modules with expressions robustly associated with age. B, C and D: modules enriched for ‘Translational elongation’, ‘Cytolysis’, and ‘DNA metabolic process’, respectively. Node’s shape and color reflect the results of the individual-gene statistic (Ui).

To test whether transcriptional changes of the 27 identified modules associate with chronological age, an expression profile for each module was constructed by determining the mean expression of the genes within a detected coexpressed PPI module per individual. As with the individual-gene analysis, we proceeded by computing the associations of the module expressions with age while adjusting for gender for each dataset separately. Only one module [Fig. 2A], enriched for ‘*T-cell activation*’, was significantly associated with age in each of the four datasets of the compendium. This module A contains genes commonly employed as markers for assessing the differentiation status of T-cell lineages, such as *CCR7*, *CD28,* and *TNFRSF7* (*CD27*). A fixed-effect meta-analysis on the expression of the different modules across the datasets showed again that the ‘*T-cell activation*’ module was most significantly associated with age (Bonferroni corrected *P* = 3.5 × 10^−38^) [see also Experimental procedures]. The consistent age association of the ‘*T-cell activation*’ module, however, raises the concern that the identified modules reflect age-related changes in the proportions of cell populations in blood, as previously reported (Derhovanessian *et al*., 2010), rather than changes in gene expression.

### T-cell activation module expression marks blood lymphocyte counts

To investigate the relation between the expression of the ‘*T-cell activation*’ module and the proportions of blood cell populations, for which we have no data in the compendium, we revisited a transcriptomic dataset on peripheral blood measured in the Leiden Longevity Study (LLS) (Passtoors *et al*., 2012) [Data S1, Supporting information]. Using the expression data of 50 middle-aged and 50, 90-year-old individuals, we first confirmed the association with age of the expression of the ‘*T-cell activation*’ module (*P* = 3.7 × 10^−5^), and subsequently observed a significant correlation between the expression of the ‘*T-cell activation*’ module and lymphocyte counts (*R*^2^ = 0.603, *P* = 1.9 × 10^−10^). These findings suggest that the previously observed age associations in the blood compendium are most probably confounded by the age-associated decline in lymphocyte counts. We also conclude that the expression of the ‘*T-cell activation*’ module could serve as a proxy for the age-associated decline in lymphocyte counts in the compendium.

### Five coexpressed PPI modules associate with age independent of T-cell activation

Based on these findings, we adapted the fixed-effect meta-analysis to reanalyze the 27 modules in the compendium while adjusting for gender as well as the expression of the ‘*T-cell activation*’ module. This revealed nine modules significantly associated with chronological age, of which five also showed a significant association without adjusting for ‘*T-cell activation*’ [Fig. 2B–F]. These five modules thus exhibit the most robust expression changes with age and include (i) a large consistently down-regulated ribosomal module (*P* = 9.4 × 10^−19^), enriched for ‘*Translational elongation*’ (*P* = 4.5 × 10^−46^); (ii) an up-regulated module containing among others several granzymes and the perforin gene (*P* = 2.9 × 10^−24^), enriched for ‘*Cytolysis*’ (*P* = 9.4 × 10^−05^); and (iii) a down-regulated module containing the *PARP1 (ADPRT)* gene (*P* = 3.1 × 10^−39^) enriched for ‘*DNA metabolic process*’ (*P* = 0.0036). The two remaining modules were both down-regulated with advancing age and lacked any significant functional enrichments (Fig. 2E,F; *P* = 3.9 × 10^−11^ and *P* = 2.5 × 10^−18^, respectively).

### Replication of coexpressed PPI modules as robust markers for aging

We conducted an independent replication study of the identified network modules as robust markers for chronological age using gene expression data from the Netherlands Twin Register and Netherlands Study of Depression and Anxiety (NTR & NESDA) consortium (*N* = 3535) (Boomsma *et al*., 2008) assayed on individuals within age range 17–79 years [Data S1, Supporting information]. An association analysis between the mean expression of a module and chronological age, adjusted for sex and the mean expression of the ‘*T-cell activation*’ module, yielded significant results for four of the five identified modules, all with directions corresponding to those found in the compendium [Table S6, Supporting information]. These results emphasize the robustness of the findings produced by our approach and confirm that the mean module expression in whole blood of module B, C, E, and F may be considered as robust markers of chronological age.

### Coexpressed PPI modules are enriched for GenAge longevity and aging genes

As a validation of the identified modules, we computed whether aging-related genes stored by GenAge (de Magalhaes & Toussaint, 2004), a database providing a comprehensive overview of aging-related genes in humans and model systems, were enriched within modules A–F (Fig. 2) [Data S1, Supporting information]. Whereas module A was supported by human derived annotations only (OR = 12.1, 95% CI 2.88–39.2, *P* = 6.95 × 10^−4^), module B was solely based on knowledge derived from model organisms (OR = 16.9, 95% CI 7.26–39.1, *P* = 2.52 × 10^−10^) [Table S7, Supporting information]. Modules D, E, and F had annotations balanced over both sources, and therefore, the significance of the joint enrichment was assessed by using a resampling approach [Data S1, Supporting information], which yielded significant enrichments for modules E (*P* = 0.016) and F (*P* = 0.0029). These findings provide additional evidence that the joint expression of these modules may play a relevant role in human aging.

### Module F associates with prospective survival at old age

To investigate whether the identified modules could potentially serve as biomarkers, we studied the microarray data assayed on 50 nonagenarian individuals from the Leiden Longevity Study (Passtoors *et al*., 2012). A left truncated Cox proportional hazard model adjusted for sex and cell counts indicates that the mean expression of module F associates with prospective survival beyond the age of 90 years (*N* = 50, *N*_death_ = 45, HR = 0.265, 95% CI 0.12–0.57, *P* = 0.001). By showing that module F associates with prospective survival at old age, we illustrate its potential biological relevance.

**Figure 3:**
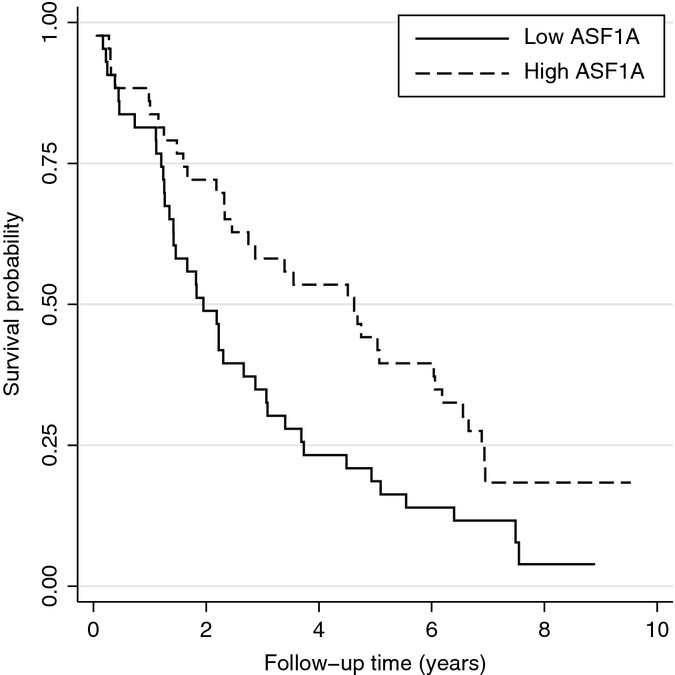
Expression of *ASF1A* associates with prospective survival in nonagenarians. High expression of *ASF1A* confers a prospective survival benefit at old age.

Interestingly, the *ASF1A* gene is part of module F and has previously been identified by our group as one of the genes that was differentially expressed in blood of members of long-lived families as compared to similarly aged controls at middle age (Passtoors *et al*., 2012). To confirm that the expression of the *ASF1A* gene in module F also associates with prospective survival at old age, we analyzed the gene expression of *ASF1A* measured with RT-qPCR in 74 nonagenarians from the Leiden Longevity Study (of which 24 overlapped with the micro-array experiment) for association with prospective survival. Because we observe a similar association (*N*_death_ = 64, HR = 0.54, 95% CI 0.34–0.85, *P* = 0.008) [Fig. 3], these results indicate that modules, of which the expression in blood is consistently associated with chronological age across various datasets, may associate with variation in lifespan, and therefore provide valid gene targets for studying relevant biological endpoints in human aging.

## Discussion

Age-associated changes in gene expression may provide meaningful leads to pathways affected by and involved in aging, though are generally difficult to detect consistently (de Magalhaes *et al*., 2009). Therefore, we constructed a large compendium of human whole blood expression studies (Goring *et al*., 2007; Emilsson *et al*., 2008; Inouye *et al*., 2010) comprising 2539 individuals on which we performed a novel integrative network-based analysis. This yielded fifteen consistently age-associated coexpressed PPI modules. Because the most significant age-associated module appeared to correlate with lymphocyte cell counts in an independent gene expression dataset, the expression of this module, enriched for ‘*T-cell activation*’, was subsequently used as a proxy for possible confounding shifts in the distribution of lymphocyte subsets. This enabled the identification of five age-associated modules [Fig. 2 Panel I and IV], including three modules enriched for ‘*Translational elongation*’, ‘*Cytolysis*’ and ‘*DNA metabolic process*’ [Fig. 2B–D]. Replication in an independent cohort confirmed these findings for four of five modules [Fig. 2B,C,E and F], underpinning the robustness of the proposed approach. The enrichments against a database for aging-related genes [Fig. 2B,E and F] emphasize the relevance of these biological findings for aging research, which is even further substantiated by the fact that the mean expression of module F associates with prospective survival at old age.

### Mitochondrion-related aging

Two of the identified modules are down-regulated with age and seem to be related to the mitochondrion, though lacking any significant functional enrichment [Fig. 2E,F]. Despite the absence of functional enrichments, both modules were significantly enriched for aging-related genes, as defined by GenAge, implying that known age-related single genes can be put into a novel biological perspective by our network approach.

Module [Fig. 2F] contains several mitochondrial factors and enzymes, like, for instance, the mitochondrial transcription termination factor *MTERF,* the *ACADM* enzyme used for fatty acid metabolism, or the mitochondrial tRNA synthetase *IARS2*, whose homolog was shown to increase lifespan upon disruption in worms (Smith *et al*., 2008). This module also includes several genes previously associated with age or age-associated diseases such as the mitotic checkpoint protein *BUB3*, previously associated with accelerated aging in mice (Baker *et al*., 2006), and the cell-cycle checkpoint protein *APPBP1* found in increased quantities in the brain affected by Alzheimer’s disease (Chen *et al*., 2003). This broad range of gene characteristics composing the module could be explained by the fact that the functionality of mitochondria is not confined to cellular energy metabolism alone, but also seems to make up an integral part of multiple cell signaling cascades including cell-cycle control and cell death (McBride *et al*., 2006).

Interestingly, module F also includes the *ASF1A* histone chaperone of which we previously have shown that its expression associates with familial longevity in the Leiden Longevity Study (Passtoors *et al*., 2012). We revisited the RT-qPCR data assayed on 74 nonagenarians and now show that the expression of *ASF1A* also associates with prospective survival. This result illustrates that modules, of which the expression in blood is consistently associated with chronological age across various datasets, may associate with variation in lifespan, and therefore provide valid gene targets for studying relevant biological endpoints in human aging.

The other mitochondrion-related module [Fig. 2E] contains the heat shock protein *HSPCA* (*HSP90)* and the mitochondrial receptor *TOMM20*, which jointly play a central role in translocating preproteins into the mitochondria (Fan *et al*., 2011). They seem to be consistently coexpressed in blood with *EIF4A2,* a eukaryotic translation initiation factor and *DDX18*, an ATP-dependent RNA helicase, of which the worm homologs were shown to extent lifespan upon disruption (Curran & Ruvkun, 2007; Smith *et al*., 2008). To summarize, this module seems to relate to aging by influencing protein translation and mitochondrial translocation efficiency.

### Age-associated limitation of protein synthesis

One of the identified modules predominantly consisted of ribosomal proteins and translation elongation factors comprising part of the ribosomal complex [Fig. 2B]. The module was significantly enriched for ‘*Translational elongation*’ and for previous findings in model organisms with respect to aging and longevity. In addition, the module was down-regulated with advancing age fitting previous observations of the aging blood transcriptome (Hong *et al*., 2008; Harries *et al*., 2011; Passtoors *et al*., 2012), which could be interpreted as an attempt of the cell to limit global protein synthesis in response to stress arising from damage accumulating throughout lifespan (Clemens, 2001). Whether caused by response to stress or other factors, the change in protein translation may be ascribed to the mTORC1 complex (Laplante & Sabatini, 2009). This complex modulates cellular growth and metabolisms by determining the balance between protein synthesis and degradation in response to nutrient availability. Inhibition of mTOR signaling through the mTORC1 complex not only inhibits protein synthesis, but also has been shown to positively affect the lifespan in various invertebrates and mammals (Johnson *et al*., 2013). Moreover, human blood transcriptome studies showed that the gene expression of mTOR pathway is down-regulated with chronological age (Harries *et al*., 2011; Passtoors *et al*., 2013) and is even associated with human familial longevity (Passtoors *et al*., 2012). Hence, a consistently down-regulated ribosomal module with advancing age corresponds with the age-associated demise of mTOR signaling. Although it is well established that mTOR signaling links to both lifespan regulation and ‘*Translational elongation*’, it remains to be determined whether down-regulation of ‘*Translational elongation*’ is causal for human aging.

### WRN-related cell-cycle checkpoint on DNA integrity

A module down-regulated with age and enriched for ‘*DNA metabolic process*’ identified in the compendium could not be replicated in the NTR & NESDA cohort [Fig. 2D]. Interestingly, this module contains the *PARP1* (*ADPRT*) gene, which directly binds to *WRN* to induce apoptosis upon oxidative stress induced DNA damage and is as such a prime suspect for Werner syndrome (von Kobbe *et al*., 2003), a premature aging disease. Furthermore, the activity of the Parp1 protein in mononuclear cells has previously been shown to positively correlate with the species-specific lifespan across 13 mammalian species (Grube & Burkle, 1992). Taken together, findings in the compendium suggest that the lowered transcription rate of *PARP1* negatively affects DNA integrity and thus lifespan, though more experiments are required to investigate this hypothesis.

### Age-associated shifts in T-cell composition

Another identified module is up-regulated with age and enriched for ‘*Cytolysis*’ [Fig. 2C]. It contains several genes used to dispatch virus-infected cells and may reflect the decreased competence for fighting infections in an early stage, caused by an age-related deterioration of the immune system, known as immuno-senescence (Pawelec & Solana, 1997). We can, however, not rule out that the age-associated expression of *GZMA*, *GZMB,* and *PRF1* that are part of this module point to an age-associated shift in T-cytotoxic cells (Derhovanessian *et al*., 2010; Napoli *et al*., 2012).

Though identified coregulated PPI modules may show extensive correlation with confounding factors, we should be careful to dismiss modules as such only. For instance, the ‘*T-cell activation*’ module [Fig. 2A], which is down-regulated with age, also contained *BNIP3*, an inhibitor of the mTORC1 complex shown to modulate lifespan in worms, flies, and mice (Johnson *et al*., 2013); and *FOXO1*, also displaying an intricate interplay with both complexes of *mTOR* (Laplante & Sabatini, 2009), and shown to extent lifespan in various invertebrates (Calnan & Brunet, 2008). Additionally, human *mTOR* signaling may play a central role in orchestrating T-cell maturation and T-cell fate decisions (Chi, 2012), and could thereby also explain the age-associated decline in lymphocytes as marked by the ‘*T-cell activation*’ module. Taken together, these examples illustrate that what is confounding the analysis of the blood transcriptome for molecular mechanisms associated to aging is subjective to debate and might even not be possible to determine given the complex interplay between the different biological levels on which aging acts.

### The proposed network approach into perspective

Network analyses have clear advantages over individual-gene analyses, as they enable the incorporation of useful prior knowledge, which can be exploited for improving the robustness of the analysis and the subsequent interpretation of the results. The improved robustness of the network approach over the individual-gene analyses was reflected by the low mutual overlap between the individual-gene results [Fig. 1] as opposed to the high concordance between the results obtained in the compendium and replication cohort. The advantages for the interpretation were clearly illustrated by the modest insights gained from the two different strategies for individual-gene analysis (*‘Glycosylation site:N-linked’*), as opposed to the detailed gene modules produced by our approach that can serve as a novel basis for further investigation into the molecular mechanisms underlying normative aging. Moreover, our approach is capable of inferring biological coherence from the data, without the explicit need of predefined functional groupings, as was shown by the enrichments of the identified modules found for genes within the GenAge database.

Though the analysis benefits from incorporating protein–protein interaction data, the type, and source clearly affect the results. To be as inclusive as possible for types and sources of PPI data, we have chosen to employ data obtained from the STRING database, which systematically collects and integrates interaction data derived from various sources for predicting functional relations between gene pairs. This choice results in a vast and comprehensive source of data. However, STRING data are not confined to physical interactions, as is the case with for instance IntAct (http://www.ebi.ac.uk/intact/) and unlike KEGG (http://www.genome.jp/kegg/), STRING data are not manually curated. For network inference, a trade-off exists between the sparsity and the quality of the employed gene–gene interactions. We made use of a threshold on the quality of reported interactions that are created by STRING by benchmarking the different interaction data sources to KEGG. Varying this threshold would affect the size and nature of the obtained coexpressed PPI modules. As the threshold determines the scale of the analysis, an interesting observation is that the results can be confounded to parts of the global network that do not necessarily overlap with the predefined known biological pathways. The latter is illustrated by the fact that some of our modules are not enriched for biological pathways and could basically be valued as a strong point of our data-driven approach.

## Conclusion

By applying a network approach to multiple blood transcriptomics datasets, we have identified five coexpression PPI modules that associate with chronological age in humans. The confirmation of most of our findings in an independent dataset underpins the robustness of our approach. The modules are significantly enriched for aging-related genes as curated by the GenAge database. This implies that these age-related single genes, in the absence of a clear understanding of their joint functioning belong to a network that finds its basis in protein–protein interactions and will serve as novel input for aging research. We reinforced the biological relevance of one of the modules by showing that it associates with prospective survival beyond 90 years in humans as was observed also for a single known age-related gene in this module (*ASF1A*). These findings collectively warrant further investigations into the biological function of module F and its potential as a biomarker for healthy aging and human longevity.

## Experimental procedures

### Creating the blood expression compendium

Analyses were based on gene expression data derived from individuals enrolled in three large cohort studies for which details on sample inclusion and employed expression protocols are provided in depth in the original publications (Goring *et al*., 2007; Emilsson *et al*., 2008; Inouye *et al*., 2010). Gene expression and accompanying phenotypic data was obtained from either the original authors or from the public data repository ArrayExpress. Data quality was stringently reexamined per dataset for the presence of outlier samples or outlier measurements and annotated to a common annotation standard (EntrezGeneID). A detailed description of the data processing and an overview on the resulting sample statistics is given in the Data S1 (Supporting information) and Table 1, respectively.

### Rank integration approach

A rank integration approach (Breitling *et al*., 2004; de Magalhaes *et al*., 2009) was used to identify genes consistently up- or down-regulated with age across multiple heterogeneous datasets. This type of meta-analysis integrates individual-gene statistics across datasets, by ranking the statistics per dataset and assessing the significance of the observed combined ranking using a Gamma distribution (Koziol, 2010) or through permutation. Gender adjusted linear fits between expression and age were used as gene statistics that were obtained by fitting the following multivariate linear regression model:

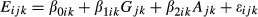
(1)where *E*_*ijk*_ is the gene expression of gene *i* for individual *j* in the *k*^th^ dataset, with 1 ≤ *i* ≤ M, 1 ≤ *j* ≤ N and 1 ≤ *k* ≤ K, where *G*_*jk*_ and *A*_*jk*_ are the gender and age of individual *j* in the *k*^th^ dataset, respectively, and where *ε*_*ijk*_ is the residual error of gene *i* for individual *j* in the *k*^th^ dataset. Genes were ranked on the regression coefficients between age and expression, *β*_*2ik*_. The rank position of gene *i* in dataset *k* is denoted by *R*_*ik*_. Ranks across the datasets were integrated per gene by computing rank product statistics as previously defined by Koziol (Koziol, 2010):

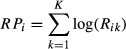
(2)

The significance of the observed rank products was assessed in two ways. Following Koziol, rank products *RP*_*i*_ were transformed using:

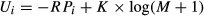
(3)

The significance of the *U*-statistics could be assessed by employing the gamma distribution (Koziol, 2010) or through permutation as described in the Data S1.

### Extracting coexpressed PPI modules

Genes were mapped to the protein–protein interaction network (STRING v9.0, http://string-db.org/), which yielded a compendium of about 81.3% of the initial set of genes (*N* = 7353) in the compendium. Ranked coexpression matrices were computed for each dataset separately by computing a correlation matrix composed of first-order partial correlations between all pairs of genes adjusted for sex and subsequently assigned a rank to each of them. A higher positive correlation resulted in a higher ranking. The ranked coexpression matrices were integrated by computing rank products as in the section on individual-gene analysis. The resulting gene–gene rank product matrix together with the PPI network matrix was subsequently used as input for the method that identifies coexpressed PPI subnetworks as described in Van den Akker *et al*. (van den Akker *et al*., 2011), see also Data S1 (Supporting information). In short, a cluster analysis on the gene–gene rank product matrix yielded coexpressed modules of genes. High confidence coexpressed genes were obtained by applying a threshold on the gene–gene rank product matrix. We obtained coexpressed PPI modules by intersecting the coexpressed gene modules with the PPI network matrix. Coexpressed PPI modules were subsequently visualized using Cytoscape [Data S1, Supporting information].

### Fixed-effect meta-analysis on module expressions across the blood compendium

Gene expression data were summarized per coexpressed PPI module for each dataset separately by taking the mean expression per individual over all genes in the module, resulting in a module expression for each dataset. Associations with age were tested for each coexpressed PPI module, by performing a fixed-effect meta-analysis across the four datasets using a first-order partial correlation between age and the module expression, computed with the controlling variable gender to adjust for sex differences. Per dataset *k,* we thus computed:

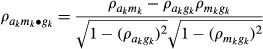
(4)where 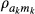 is the correlation between age and the expression of the *n*^th^ module across individuals of the *k*^th^ dataset; 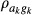 is the correlation between age, and gender across individuals of the *k*^th^ dataset and 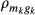 is the correlation between expression of the *m*^th^ module and gender of individuals in the *k*^*th*^ dataset. To correct for multiple controlling variables, higher order partial correlations were computed by repeatedly computing first-order partial correlations as described above. The function *metacor* of R package *meta* was used for integrating and testing the meta correlation statistic between age and module expression across the four datasets using default settings. Modules with significant correlations (Bonferroni corrected *P*-value ≤ 0.05) were considered age dependent.

## Supporting Information

**Fig. S1** Overview of detected consistently coexpressed PPI modules.

**Table S1** Results of 26 significantly age-associated genes in all four datasets.

**Table S2** Results of the individual-gene rank product test.

**Table S3** Enrichment analyses on the 26 significantly age-associated genes in all four datasets.

**Table S4** Enrichment analyses on 195 significant genes obtained with an individual-gene rank product test.

**Table S5** Gene enrichment analyses using DAVID on the 27 consistently coexpressed PPI modules counting at least 5 genes.

**Table S6** Replication of the identified modules as robust markers of chronological age in the NTR & NESDA cohort.

**Table S7** GenAge enrichment analyses of identified coexpressed PPI modules.
