# Supplementary material for: Meta-analysis on blood transcriptomic studies identifies consistently coexpressed protein–protein interaction modules as robust markers of human aging
Source: Aging Cell. 2013 Nov 19;13(2):216–25. doi: 10.1111/acel.12160 (PMC4331790; doi:10.1111/acel.12160)
Supplement: Supplementary file 9 — Data S1 Supplemental methods. [file acel0013-0216-sd9.doc]

**Supplemental methods [SM]**

**SM 1: Data preprocessing**

When missing measurements were reported for a dataset, a filtering on success rates per sample and probe was performed to remove problematic arrays or failed measurements across samples. To check for the presence of substructure across samples, density plots of whole sample expression data, scatter plots using the first four principal components, and clustering analyses on whole sample correlations were employed.

Data of Göring et al. (SAFHS) showed no signs of substructure and passed all set quality criteria, yielding a dataset of 1,240 samples. Data of Emilsson et al. (IFB) was measured in two large batches and was treated as separate datasets. Replicate samples between both datasets were removed from the largest batch (IFB_B). On recommendation of the original authors, expression values ≤ -2 and ≥ 2 were set to missing before filtering on success rates per probes. Samples with success rates below 98% (more than 2% of the data was missing) or with aberrant density distributions were removed yielding two datasets of respectively 411 (IFB_A) and 434 (IFB_B) samples. Probes with success rates below 98% per dataset were removed and remaining missing values were imputed using nearest neighbor averaging implemented in the function *impute.knn* of R package *impute* with default settings.

Preprocessed data of Inouye et al. (DILGOM) showed some grouped outliers in the PCA analysis [Fig SM1]. Upon inspection of the raw data, this batch effect was also visible in the density plots of whole sample expression data [Fig SM2 and SM3], leading to the decision to exclude samples within the smallest batch and to redo the processing of the raw data. Raw data was measured in replicates and the remaining 454 samples were quantile normalized per replicate using the R function *normalizeBetweenArrays* of the package *limma*. Data of the 2^nd^ replicate was used for further analyses as the rankings of gene-gene correlations across these chips were most similar to those observed in SAFSH, IFB_A and IFB_B [Fig SM4].

**SM 2: Network Inference across a collection of heterogeneous expression datasets**

Modules were identified as previously described in Van den Akker et al. with some adjustments for estimating an integrated correlation matrix using all four datasets. Expression data was first mapped to the PPI network (STRING v9.0 ) by using the gene2ensembl converter downloaded from NCBI’s FTP server. An integrated gene-gene correlation matrix was computed using data of those genes that mapped to the PPI network only [SM 2.1]. Co-expressed PPI sub-networks were then identified as originally described using two types of evidence: PPI data and expression correlation measures between pairs of genes [SM 2.2].

**SM 2.1: Computation of the integrated correlation matrix**

To cope with heterogeneity between the studies we chose to integrate the correlations matrices between genes over different studies using a rank-based approach. To this end, we first computed gene-gene correlation matrices for each dataset separately. Then let ***C*** be the *M × M* sample correlation matrix holding the gender adjusted correlations between gene *p* and *q* for the *k*^th^ dataset computed over all samples *N_k_*. Ranking of these correlation matrices yields a set of ranking matrices denoted by ***RC***. A gene-gene rank product matrix ***RPC*** denoting the integrated rankings of gene-gene correlations between gene *p* and *q* across all datasets can now be computed using:

$RPC_{pq}=\sum_{k=1}^{L} \log(RC_{pqk})$ (S1)

**SM 2.2: Identifying consistently co-expressed PPI modules**

The resulting integrated gene-gene correlation matrix was hierarchically clustered (average linkage) and was cut at height 1-*T_COR_* yielding disjoint sets of co-expressed modules of genes. Functional groupings were found by maintaining the edges between genes that had a PPI confidence score >= *T_PPI_* and a gene-gene correlation >= *T_COR_*. More formally: Let matrix ***P*** be the matrix with reported protein-protein interactions, with ***P****_pq_* ranging from 0 to 999 indicating the confidence score in the reported functional interaction between gene *p* and *q*. Let ***CM****_pq_***^*T^** be the binairy matrix after applying *T_COR_* on ***CM****_pq_***^*^** where ***CM****_pq_***^*T^** = 1 indicates sufficient and ***CM****_pq_***^*T^** = 0 indicates insufficient correlation between genes *p* and *q* respectively. Likewise, let ***P***^T^ be the equivalent of ***CM****_pq_***^*T^** when *T_PPI_* is applied on ***P***. Let matrix ***G****_pq_* be a binary *M × M* matrix holding the indices for module co-membership between gene *p* and *q* obtained by the clustering.

Functional groupings within the co-expressed modules of genes ***S_pq_*** are then found by:

$S_{pq}=G_{pq}CM_{pq}^{*T}P_{pq}^{T}$ (S2)

The thresholds on the PPI confidence: TPPI and correlation TCOR were determined as follows. First the TPPI was set to a value of 400 which is indicated by the STRING database as “medium confidence”. This corresponds to 104,948 gene-gene pairs between the 7,353 genes that could be mapped to the PPI network. To balance the influence of the two types of evidence for sub-network construction, the threshold for the gene-gene correlations: TCOR was set to match the prior probability of two random genes to pass the TPPI threshold (p = 0.00388), resulting in TCOR = 0.429.

**Figures in Supplemental Methods**

**Fig SM1:** Using the function prcomp of R, the first four principal components were computed over the samples and used for pair wise scatter plots. The first row depicts the samples projected on the first principal component (x-axis) versus respectively the second, third and fourth principal component (y-axis). A flock of outliers is clearly visible in the first versus fourth component scatter plot.

**Fig SM2:** Whole array density distributions on the raw data clearly indicate the presences of two batches. Distributions of arrays in the right group coincided with the outlier samples in fig SM1.

**Fig SM3:** A histogram and density distribution of the most frequently observed expression value per array indicates the relative sizes of the two batches and potential decision borders for splitting the data. Arrays above a value of 7.5 (x-axis) were discarded and in general were pairs of replicate arrays belonging to the same sample.

**Fig SM4:** Raw data was normalized per replicate. Since our aim is to infer gene regulatory networks using gene-gene correlations across multiple datasets, we inspected which of the two replicate datasets showed gene-gene correlations most similar to those observed in the other datasets. For this purpose, we computed gene-gene correlation matrices for all datasets, the two replicate datasets of Inouye et al. and for two other large datasets that were expected to have a different gene-gene correlation structure from blood (EmilssonAdipose and Schadt . Similarities between resulting matrices were determined by using the Spearman’s rank correlation. Two heatmaps were drawn to visualize these pair wise similarities between datasets with each using a different replicate dataset of Inouye . Whereas the correlation structure in the first replicate (left) is unlike any other, the second replicate (right) clusters with the other blood datasets (Goring , Emilsson_A and Emilsson_B ). We therefore continued our analysis using the second replicate created by Inouye et al..

**References in Supplemental Methods**

1. Goring HH, Curran JE, Johnson MP, Dyer TD, Charlesworth J, et al. (2007) Discovery of expression QTLs using large-scale transcriptional profiling in human lymphocytes. Nat Genet 39: 1208-1216.
2. Emilsson V, Thorleifsson G, Zhang B, Leonardson AS, Zink F, et al. (2008) Genetics of gene expression and its effect on disease. Nature 452: 423-428.
3. Inouye M, Silander K, Hamalainen E, Salomaa V, Harald K, et al. (2010) An immune response network associated with blood lipid levels. PLoS Genet 6.
4. van den Akker EB, Verbruggen B, Heijmans BT, Beekman M, Kok JN, et al. (2011) Integrating protein-protein interaction networks with gene-gene co-expression networks improves gene signatures for classifying breast cancer metastasis. J Integr Bioinform 8: 188.
5. Szklarczyk D, Franceschini A, Kuhn M, Simonovic M, Roth A, et al. (2011) The STRING database in 2011: functional interaction networks of proteins, globally integrated and scored. Nucleic Acids Res 39: D561-568.
6. Schadt EE, Molony C, Chudin E, Hao K, Yang X, et al. (2008) Mapping the genetic architecture of gene expression in human liver. PLoS Biol 6: e107.
